# Ecological and human health risk assessment of heavy metal(loid)s in agricultural soil in hotbed chives hometown of Tangchang, Southwest China

**DOI:** 10.1038/s41598-022-11397-0

**Published:** 2022-09-01

**Authors:** Cang Gong, Shunxiang Wang, Dewei Wang, Haichuan Lu, Hang Dong, Jiufen Liu, Buqing Yan, Liang Wang

**Affiliations:** 1 Civil-Military Integrated Geological Survey Center of China Geological Survey, Chengdu, China; 2 Chengdu Geological Survey Center of China Geological Survey, Chengdu, China; 3 Natural Resources Comprehensive Survey Command Center of China Geological Survey, Beijing, China

**Keywords:** Ecology, Environmental sciences

## Abstract

To determine the heavy metal(loid)s (HMs) contamination of agricultural soil in hotbed chives hometown of Tangchang, 788 topsoil samples were collected and analyzed for their heavy metal(loid)s concentration. The index of geo-accumulation (I_geo_), pollution index (PI) and potential ecological risk index (EI_*i*_) were used to assess the degree of pollution. Correlation analysis and principal component analysis (PCA) were used to determine the sources of soil HMs. Human health risks estimated with hazard index (HI) and carcinogenic risk (CR) indices based on ingestion, inhalation and dermal exposure pathways for adults and children. The mean values of Cd, Hg, As, Pb, Cr, Cu, Ni and Zn were 0.221, 0.155, 9.76, 32.2, 91.9, 35.2, 37.1 and 108.8 mg kg^−1^, respectively, which did not exceed the threshold values of the risk screening value for soil contamination. The potential ecological risk of soil heavy metal(loid)s was low level and there was no significant human health risk. Based on PCA, Pb and Hg may originate from transportation and atmospheric deposition, Zn, Cr and Ni may originate from natural sources and industrial activities, and Cu and Cd may originate from agricultural activities. Overall, from the perspective of HMs content, the soil quality in this study area was at a clean level. This study provides a reference and a basis for formulating effective measures to prevent and control HMs enrichment in agricultural soils.

## Introduction

In recent years, soil heavy metal(loid)s (HMs) pollution has become a worldwide environmental problem. HMs in agricultural soils have been widely concerned because they are related to the quality and safety of agricultural products and human health^1–4^. According to the China’s national soil pollution survey in 2014, the total over standard rate of soil in China was 16.1%, and the proportions of slight, mild, moderate and severe pollution sites were 11.2%, 2.3%, 1.5% and 1.1%, respectively, the over standard rate of point for Cd, Hg, As, Cu, Pb, Cr, Zn and Ni were 7.0%, 1.6%, 2.7%, 2.1%, 1.5%, 1.1%, 0.9% and 4.8% respectively, the over standard rate of cultivated soil points was 19.4%, and the over standard points of inorganic pollutants accounted for 82.8% of all the over standard points. Due to the carcinogenic and toxic characteristics of some HMs such as As, Cd, Cr and Ni, any contamination of arable soil with these HMs should be considered carefully. Although trace amounts of Cu, Zn, Mn, Co and other metal elements are necessary for human and other organisms, but excess uptake of these metals can impair the physiological function of various body organs^5–7^. For Cd, As, Cr and etc. it has been demonstrated that, they are highly toxic, non-degradable, and can be biologically accumulated in humans and animals body, thus result in irreversible health effects^8^. The natural activities of the parent materials and anthropogenic activities of agricultural inputs, industrial discharge, atmospheric deposition, and vehicle exhaust can cause potential contamination with heavy metal(loid)s to soil, it not only leads to the decline of crop yield and quality, but also harms human health through the food chain^9–13^.

Hotbed chive is a kind of characteristic vegetable which is produced by the dull softening cultivation of the persistent root of Chinese chive. It is rich in vitamins, protein, minerals and volatile sulfide, and has high nutritional and health value. However, in the growth process of hotbed chive, it is easy to be affected by As, Cd and other HMs. When the concentration of As, Cd, Cu and other HMs in the soil exceeds a certain value, the germination and rooting of hotbed chive are obviously inhibited, and then the yield of hotbed chive is affected^14,15^. To a certain extent, eating hotbed chive with excessive heavy metal(loid)s can cause human health risk. Therefore, it is urgent to strengthen the risk assessment of soil heavy metal(loid)s pollution in hotbed chive producing areas.

Tangchang town is the famous hometown of hotbed chive in China. The "Tangyuan hotbed chive " produced is a product protected by national geographical indications. The planting scale of hotbed chive in Tangchang town reaches 10,860 acres and the output reaches 27,000 tons. It is the largest production base of hotbed chive in Southwest China, and products are exported to Hong Kong, Macao and other countries. With the rapid expansion of urbanization, the rapid development of industrial and mining enterprises and the extensive use of agricultural substances such as pesticides and chemical fertilizers, the soil environment of Chengdu Plain has changed to varying degrees, which directly affects the content of HMs in agricultural products such as TangChang hotbed chive in the west of Chengdu Plain. Liu et al.^16^ reported that 18.6% of Chengdu Plain was polluted by HMs to varying degrees. Li et al.^17^ showed that the content of Cd in most districts and counties of Chengdu Plain increased in varying degrees compared with 1982. Liu et al.^18^ investigated about 60,000 km^2^ of Chengdu Plain and found that there were serious excessive HMs such as Cd, Pb, Hg and As in the topsoil. However, to the best of our knowledge, there are no relevant reports on the distribution of HMs in TangChang soil and their associated risks to humans and ecosystems, there is a lack of detailed data to explain whether the content of HMs in soil in this area maintains a clean level. Therefore, in order to ensure the quality and yield of hotbed chive, it is very necessary to carry out a comprehensive risk assessment of soil HMs in this area. In the present study, our main objectives were to: (1) to assess the enrichment and pollution levels of HMs in soils, (2) identify the main source of HMs, (3) describe distributions and trend of soil HMs pollution, (4) evaluate the potential ecological risk and human health risk of HMs.

## Material and methods

### Study area

**Figure 1:**
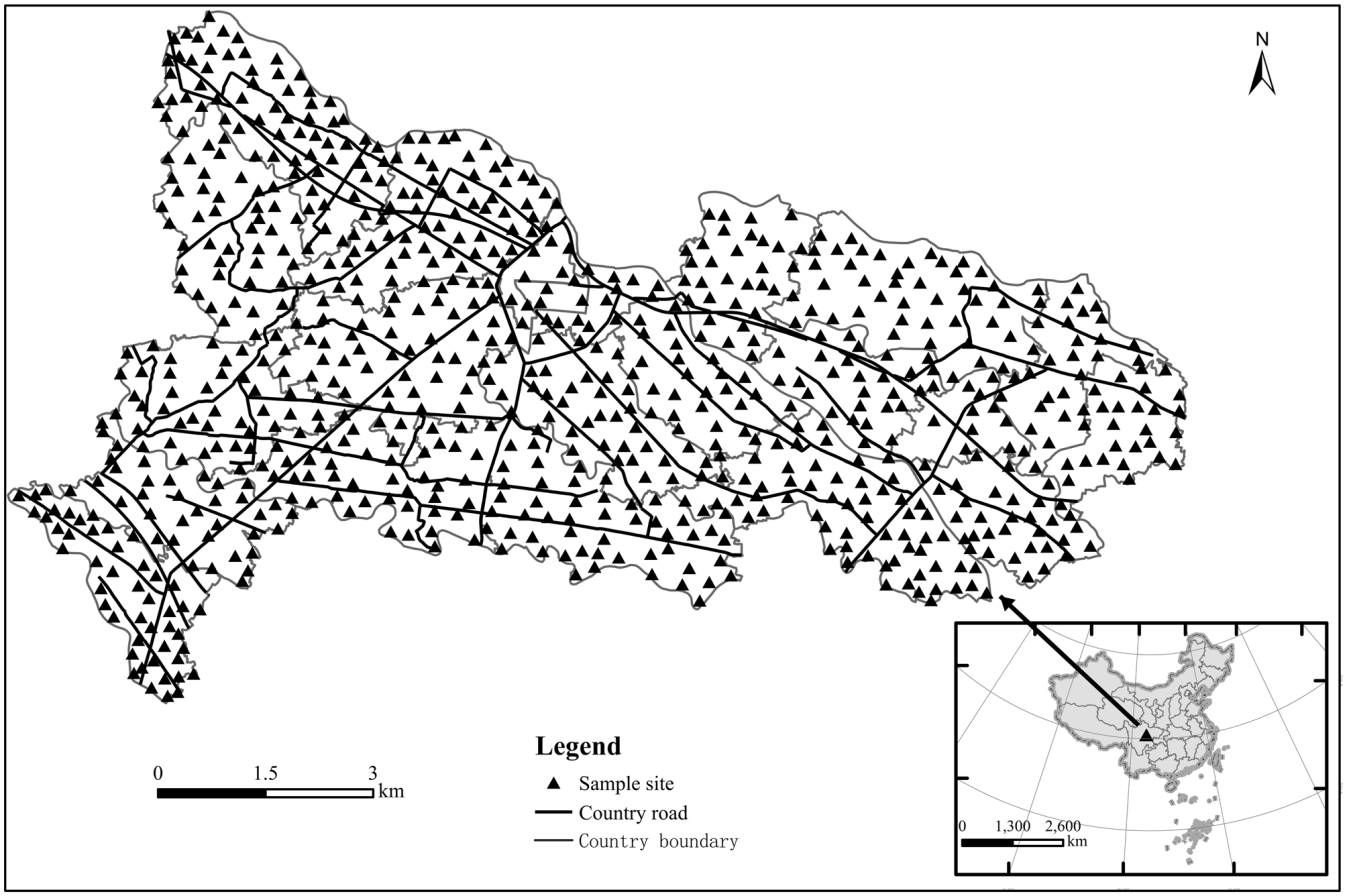
Locations of study area and sampling sites. (Map were generated with software ArcMap10.8 http://www.esri.com/).

As shown in Fig. 1, TangChang town is located in the hinterland of Chengdu Plain in southwest China, about 40 km away from the urban area of Chengdu, 103°44′44″–103°54′51″ E, 30°52′22″–30°56′20″ N. It covers an area of 74.6 km^2^ and 95% of the cultivated land. It has a subtropical monsoon humid monsoon climate with an average annual temperature of 15.7 °C, an average annual rainfall of 972 mm and an average annual sunshine time of 1280.9 h. The soil is fertile and belongs to purple alluvial soil. Baitiao River, Baimu River, Xuyan River and Zouma river flow through the town. It is an important vegetable basket base in Chengdu, the largest hotbed chives production base in Southwest China, and the “hometown of hotbed chives” in China.

### Sample collection and measurement

Field sampling was performed in April 2021. The sample collection was done based on specification of land quality geochemical assessment (DZ/T 0295-2016), a total of 788 topsoil (0–20 cm) samples were collected. The sampling locations are shown in Fig. 1. At the same time, to improve the representativeness of the soil samples, five sub-samples were collected from a 20–50 m region around each sampling point by X-type sampling method and mixed into one sample, the sampling sites were located using a portable GPS. The samples were stored in sealed polyethylene bags until they were transported to the laboratory and were naturally air-dried for one week, removed other debris, and then sieved through a 10-mesh plastic sieve for later use.

In order to determine heavy metals contents of Cu, Pb, Zn, Cr, Ni and Cd, 0.1000 g soil samples were weighed accurately, placed into a PVC digestion vessel, and then digested with 10 ml HNO_3_-HCl-HClO_4_-HF (excellent grade). The concentrations of Cu, Pb, Zn, Ni and Cd were measured by inductively coupled plasma mass spectrometry (ICP-MS, NexION350X, USA)^19^, Cr were measured by inductively coupled plasma atomic emission spectrometry (ICP-AES, Avio500, USA). For As and Hg, 0.2500 g soil samples were weighed, placed into a tall beaker, and by water bath digestion with 10 ml aqua regia, the contents were measured by atomic fluorescence spectrophotometry (AFS, AFS8500, CHN)^20,21^. Soil pH was measured by ion selective electrode method^22^, the content of total potassium (TK) and total phosphorus (TP) was measured by X-ray fluorescence (XRF, Axios mAX, NLD)^23^, soil total nitrogen (TN) was measured by elemental analyzer method^24^, and the content of organic matter (OM) was measured by volumetric method^25^. In the process of measurement, duplicate tests were carried out, and quality control procedures were conducted using state first-level standard materials (GSS5, 7–9, 20, 23, 24, 28). The Primary original qualified rate of all state first-level standard materials not lesser than 98%. The limit of detection (LOD) values for Cu, Pb, Zn, Ni, Cd, Cr, As and Hg were obtained to be 1, 2, 4, 2, 0.01, 5, 0.5 μg g^−1^ and 0.0003 μg g^−1^, respectively. There were 64 samples for duplicate tests, the qualified rate of duplicate tests for HMs were 100%, except Hg was 93.8%.

### Index of geo-accumulation

To assess the degree of contamination and compare concentration of different HMs in soil samples, the index of geo-accumulation (I_geo_) was used, defined as follows^2^:

1 $$ {\text{I}}_{{{\text{geo}}}} = {\text{log}}_{{2}} \left( {\frac{{C_{i} }}{{K \times B_{i} }}} \right) $$

where C_*i*_ is the concentration of *i* element at the sampling site (mg kg^−1^); B_*i*_ is the geochemical background concentration (mg kg^−1^) of *i* element in Chengdu^26^, K is a constant value and equals to 1.5. There are seven classes based on I_geo_ value: ≥ 5, 4–5, 3–4, 2–3, 1–2, 0–1, < 0, representing extremely contaminated, strongly to extremely contaminated, strongly contaminated, moderately to strongly contaminated, moderately contaminated, uncontaminated to moderately contaminated, uncontaminated, respectively.

### Pollution index and synthetic pollution index

In order to assess the level of HMs pollution in the soil, a single factor pollution index (PI) and a synthetic pollution index (SPI) were calculated:

2 $$ {\text{PI}} = \frac{{C_{i} }}{{S_{i} }} $$

3 $$ {\text{SPI }} = \sqrt {\frac{{\left( {\frac{{C_{i} }}{{S_{i} }}} \right)_{max} + \left( {\frac{{C_{i} }}{{S_{i} }}} \right)_{min} }}{2}} $$

where PI is the pollution index of each element and SPI is the synthetical score of each heavy metal(loid)s to the composite pollution. S_*i*_ is the evaluation standard of the *i* element, and the national control thresholds were chosen as the standard (Table 1). elements. There are five pollution categories based on PI and SPI values: < 0.7, 0.7–1, 1–2, 2–3, ≥ 3, representing safety, alert, low pollution, moderate pollution, and severe pollution, respectively^27^.

**Table 1:** Statistical summary of HMs concentrations (mg kg^−1^) in soil, and TN, TP, TK, OM (g kg^−1^) and soil pH.

Elements	Mean	S.D	Median	Min	Max	CV%	Background values^a^	Threshold values^b^			
								A	B	C	D
Cd	0.221	0.069	0.210	0.082	0.83	31.05	0.25	0.3	0.3	0.3	0.6
Hg	0.155	0.090	0.130	0.022	0.88	58.13	0.08	1.3	1.8	2.4	3.4
As	9.76	2.08	9.43	4.17	18.0	21.35	9.11	40	40	30	25
Pb	32.2	5.0	32.3	19.8	90.3	15.42	30.3	70	90	120	170
Cr	91.9	10.0	92.3	61.7	264	10.87	78	150	150	200	250
Cu	35.2	21.1	34.4	18.5	607	59.93	28.1	50	50	100	100
Ni	37.1	3.7	37.2	23.6	56.6	10.07	33.5	60	70	100	190
Zn	108.8	62.9	106.0	55	1820	57.80	82.2	200	200	250	300
pH	6.17	1.01	5.96	4.16	9.04	16.40	6.14	–	–	–	–
TN	1.33	0.26	1.30	0.70	2.40	19.6	–	–	–	–	–
TP	1.16	0.79	1.11	0.22	20.8	68.14	–	–	–	–	–
TK	23.6	2.4	24.3	10.3	28.0	10.15	–	–	–	–	–
OM	17.7	5.1	17.6	2.4	43.8	28.62	–	–	–	–	–

^a^The background values of soil metals for Chengdu^26^.

^b^The risk screening values for soil contamination (GB 15618-2018) (MEEC, 2018), A: pH ≤ 5.5, B: 5.5 < pH ≤ 6.5, C: 6.5 < pH ≤ 7.5, D: pH > 7.5.

### Potential ecological risk assessment

The potential ecological risk index (EI_*i*_) and comprehensive potential ecological risk index (RI) were proposed by Lars^28^. Which were employed to assess the degree of ecological risk based on the toxicity, concentrations, characteristics and environmental behavior of HMs.

4 $$ {\text{EI}}_{i} = {\text{T}}_{i} \frac{{C_{i} }}{{B_{i} }} $$

5 $$ {\text{RI}} = \mathop \sum \limits_{i = 1}^{n} EI_{i} $$

where EI_*i*_ is the potential ecological risk factor of individual HMs, T_*i*_ is the toxic response factor of single HMs. In this study, the T_*i*_ values of Hg, Cd, As, Cu, Ni, Pb, Cr, and Zn were 40, 30, 10, 5, 5, 5, 2, and 1, respectively^29^. The EI_*i*_ is defined in five categories as: low (< 40), moderate (40–80), considerable (80–160), high (160–320) and very high (≥ 320). Five categories are defined for RI: low (< 150), moderate (150–300), considerable (300–600), very high (600–1200), and dangerous (≥ 1200), representing^30^.

### Health risk assessment

The human health risk assessment of HMs in soils is widely carried out using exposure evaluation. In this study, three exposure pathways associated with soil HMs were explored: ingestion (ADD_ing_), inhalation (ADD_inh_) and dermal contact (ADD_derm_). The estimated average daily intake of HMs (ADI, mg kg^−1^ day^−1^) via ADD_ing_, ADD_inh_ and ADD_derm_ for both adults and children was as follows^31,32^.

6 $$ ADD_{ing} = \frac{{C_{i} \times IngR \times EF \times ED}}{BW \times AT} \times 10^{ - 6} $$

7 $$ ADD_{inh} = \frac{{C_{i} \times InhR \times EF \times ED}}{BW \times AT \times PEF} \times 10^{ - 6} $$

8 $$ ADD_{derm} = \frac{{C_{i} \times SA \times AF \times ABS \times EF \times ED}}{BW \times AT \times PEF} \times 10^{ - 6} $$

Detailed parameter values of IngR, InhR, EF, ED, BW, AT, PEF, SA, AF and ABS are listed in Table 2.

**Table 2:** Exposure parameters, reference dose (*RfD*) and slope factor (*SF*) of HMs.

Parameters	Value
IngR (ingestion rate of soil) (mg day^−1^)	100 (adults) and 200 (children)
InhR (inhalation rate) (m^3^ day^−1^)	14.5 (adults) and 7.5 (children)
EF (exposure frequency) (day year^−1^)	350
ED (exposure duration) (year)	25 (adults) and 6 (children)
BW (average body weight) (kg)	56.8 (adults) and 15.9 (children)
AT (average exposure time) (days)	ED × 365 (non-carcinogenic risk) and 26,280 (carcinogenic risk)
SA (surface area of skin) (cm^2^)	5700 (adults) and 2800 (children)
AF (skin adherence factor) (mg (cm^2^ h)^−1^)	0.2
PEF (Emission factor) (m^3^ kg^−1^)	1.36 × 10^9^
ABS (dermal absorption factor) (unitless)	0.001
*RfD* for ingestion (unitless)	As (3.00 × 10^−4^), Cd (1.00 × 10^−3^), Cr (3.00 × 10^−3^), Cu (4.00 × 10^−2^), Hg (3.00 × 10^−4^), Ni (2.00 × 10^−2^), Pb (3.50 × 10^−4^) and Zn (3 × 10^−1^)
*RfD* for dermal absorption (unitless)	As (1.23 × 10^−4^), Cd (2.5 × 10^−5^), Cr (6 × 10^−5^), Cu (1.20 × 10^−2^), Hg (2.1 × 10^−5^), Ni (5.40 × 10^−3^), Pb (5.25 × 10^−5^) and Zn (6 × 10^−2^)
*RfD* for inhalation (unitless)	As (3.0 × 10^−4^), Cd (1.00 × 10^−5^), Cr (2.86 × 10^−5^), Cu (4.02 × 10^−2^), Hg (8.57 × 10^−5^), Ni (2.06 × 10^−3^), Pb (3.52 × 10^−5^) and Zn (3.0 × 10^−1^)
*SF* for ingestion (unitless)	As (1.50 × 10^0^), Cd (6.10 × 10^0^), Cr (5 × 10^−1^) and Pb (8.5 × 10^−3^)
*SF* for dermal absorption (unitless)	As (1.50 × 10^0^) and Cd (6.10 × 10^0^)
*SF* for inhalation (unitless)	As (1.51 × 10^1^), Cd (6.30 × 10^0^), Cr (4.20 × 10^1^), Ni (8.40 × 10^−1^) and Pb (4.20 × 10^−2^)

Hazard index (HI, unitless) and hazard quotient (HQ, unitless) are usually used to indicate the risk level of human exposure for non-carcinogenic contaminants. Carcinogenic risk (CR, unitless) is usually used to indicate the probability of inducing cancer when humans are exposed for carcinogenic contaminants. When the contaminants enter through multiple pathways of exposure, assuming that there is no antagonistic effect or cooperative effect between the contaminants, the HI for all exposure pathways and the CR can be calculated as follows^1^:

9 $$ {\text{HI}} = \mathop \sum \limits_{i = 1}^{n} HQ_{i} = \mathop \sum \limits_{i = 1}^{n} \frac{{ADD_{i} }}{{RfD_{i} }} $$

10 $$ {\text{CR}} = {\text{ADD}} \times {\text{SF}} $$

where SF is the cancer slope factor of the HMs via ADD_ing_, ADD_inh_ and ADD_derm_ of soil particles (mg kg^−1^ day^−1^) and RfD is the reference dose of the HMs through ADD_ing_, ADD_inh_ and ADD_derm_ of soil particles (mg kg^−1^ day^−1^). Detailed values are listed in Table 2. Generally, HQ or HI is ≤ 1, it is considered that there are no significant risks of noncarcinogenic effects. For CR, the acceptable risk for regulatory purposes is in the range from 1.0 × 10^−6^ to 1.0 × 10^−4^.

### Data analysis

Descriptive statistics, such as mean, standard deviation, median, maximum, minimum and coefficient of variation were used to characterize the contents of HMs in soil samples. Microsoft Excel 2010 and SPSS 26 and Origin 2019b were used to process the experimental data, perform for correlation analysis of HMs concentrations, and test significance analysis for soils properties and biomarker responses at P < 0.05 and P < 0.01 levels. Adobe Origin 2019b was also taken to draw graphics.

## Results and discussion

### Soil physical–chemical properties and HMs concentrations

The soil physical–chemical properties and HMs concentrations are summarized in Table 1. The soil mean pH value was 6.17 and ranged from 4.16 to 9.04 in different sites. The samples sites of level for pH ≤ 6.0(acidic soil), 6.0 < pH ≤ 7.5(Neutral soil) and pH > 7.5(alkaline soil) were 51.5%, 36.4% and 12.1%, respectively. The average content of TN, TP and TK were 1.33 g kg^−1^, 1.16 g kg^−1^ and 23.6 g kg^−1^, and ranged from 0.7 g kg^−1^ to 2.4 g kg^−1^, 0.22 g kg^−1^ to 20.8 g kg^−1^ and 10.3 g k g^−1^ to 28 g kg^−1^, respectively.

The mean concentration of Cd, Hg, As, Pb, Cr, Cu, Ni and Zn were 0.221, 0.155, 9.76, 32.2, 91.9, 35.2, 37.1 and 108.8 mg kg^−1^. Except Cd, the average concentration of Hg, As, Pb, Cr, Cu, Ni and Zn exceeded 93.8%, 7.1%, 6.3%, 17.8%, 25.3%, 10.7%, and 32.4% the soil background values for Chengdu, respectively, which indicates that HMs are enriched to a certain extent in soil. The CV of the HMs in the agricultural soils increased in the order Ni(10.1%), Cr(10.9%), Pb(15.4%), As(21.4%), Cd(31.1%), Zn(57.8%), Hg(58.1%) and Cu(59.9%). The exceptionally high variability of Cu, Hg and Zn indicates that these metals differed greatly with respect to different sites, and the existence of abnormally high values is the main reason that the CV was high. It further indicates that Cu, Hg and Zn may be affected by external interference factors. The mean concentration of all HMs in soil were below the risk screening values for soil contamination (GB 15618-2018) (MEEC, 2018), however, the results showed that in 76, 1, 1, 6 and 2 of sample sites the level of Cd, Pb, Cr, Cu and Zn exceeded the risk screening values.

### Assessment of heavy metal(loid)s pollution

#### Index of geo-accumulation

**Figure 2:**
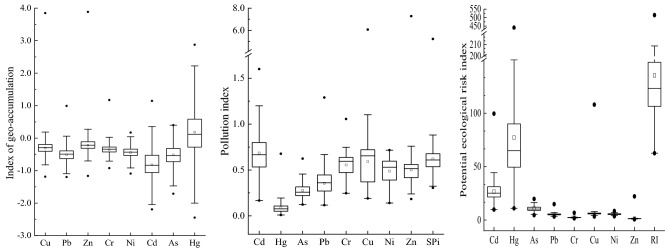
Indexes of geo-accumulation, pollution indexes and potential ecological risk indexes of HMs in study aera. Circles at the top and bottom of box plots correspond to the maximum and minimum values, respectively. The square in the box plot is the average value. Horizontal lines at the top, middle, and bottom of the box plot correspond to 75% percentile, median, and 25% percentile, respectively.

The index of geo-accumulation of HMs in the soil in the study area are shown in Fig. 2. In a descending order of magnitude of I_geo_ mean value, the eight elements were as follows: Hg(0.18) > Zn(-0.22) > Cu(-0.30) > Cr(-0.36) > Ni(-0.45) > Pb(-0.51) > As(-0.52) > Cd(-0.82), indicating that the soil HMs Zn, Cu, Cr, Ni, Pb, As and Cd in the study area were generally in a no contamination according to the defined classes, while Hg was in uncontaminated to moderately contaminated.

The I_geo_ values for Zn, Cu, Cr, Ni, Pb, As and Cd in more than 90% of samples were less than zero, only a few outliers in the soil were classified as moderately contaminated or worse. Which meaning point pollution at those sample sites. However, the I_geo_ values for Hg in 47.34%, 40.61%,10.66% and 1.40% of samples were belonged to < 0, 0–1, 1–2 and 2–3, respectively, 52.66% sample sites were contaminated moderate to strongly. It means that there were non-point source pollution sources of Hg in the study area. Relevant studies have pointed out that the overall distribution trend of soil Hg content in Chengdu Plain is relatively high in the north, which is mainly affected by geological structure, domestic pollution of urban residents and pollutant emission of industrial enterprises^17^.

#### Pollution index

The PI and SPI of soil HMs are drawn in Fig. 2. The PI average value of Cd, Hg, As, Pb, Cr, Cu, Ni and Zn were 0.69, 0.09, 0.28, 0.36, 0.56, 0.59, 0.49 and 0.50, and the ranging from 0.17 to 1.60, 0.01 to 0.68, 0.12 to 0.62, 0.12 to 1.29, 0.25 to 1.06, 0.19 to 6.07, 0.14 to 0.72 and 0.18 to 7.28, respectively. The PI value for Cd in 55 sample sites, for Cu in 5 sample sites, and for Pb, Cr and Zn in one site were belong to 1.0–2.0, indicating low pollution according to the defined classes. However PI value for Cu(6.07) and Zn(7.28) in one site were higher 3, belong to the severe pollution. It may be because the sampling point was close to the industrial area. Although the I_geo_ mean value of Hg was the largest, its PI average value was the lowest. Because of different research perspectives and different reference indicators, the results were inconsistent^33^.

The SPI is usually applied to evaluate the overall status of HMs contamination. The mean SPI value was 0.62, ranging from 0.31 to 5.22 in Tangchang agricultural soil. In our study, 98.23% of soil samples were not polluted with HMs as 1.52% of samples within low pollution and 0.25% of the samples had a severe pollution level. In general, the soil quality in the study area is generally at a clean level.

### Potential ecological risk assessment

The EI_*i*_ values for each HMs and the RI are shown in Fig. 2. The EI_*i*_ values of As, Pb, Cr, Ni and Zn in all sample sites were less than 40, meaning a low potential ecological risk with these HMs. For Cu, except that the EI_*i*_ in one sample was 108, which belongs to considerable potential ecological risk, the other samples were less than 40. For Cd and Hg, 93.4% and 9.5% of soil samples were in low potential ecological risk category, respectively. Significantly, 6.5% of samples for Cd and 55.5% for Hg were in moderate potential ecological risk categories, and one sample for Cd and 29.4% for Hg were in very high potential ecological risk categories. Especially EI_*i*_ of two soil samples for Hg were high than 320, belongs in dangerous potential ecological risk categories. In this study area the RI mean value was 135.4, and ranging from 62.6 to 514.6. In all samples 76.4% had a low potential ecological risk of HMs, 22.1% had a moderate risk and 1.5% had a considerable risk.

According to the calculation results of I_geo_ and EI_*i*_, it is found that the results of the two methods were consistent and different. Such as the I_geo_ and EI_*i*_ of Hg were the largest, however, the I_geo_ of Cd is the smallest, but its EI_*i*_ was the second highest. The reason may be that the I_geo_ focuses on the enrichment of exogenous HMs, on this basis, the EI_*i*_ focuses more on the potential effects of toxic effects of HMs. In the EI_*i*_, the toxicity coefficients of Hg and Cd were the largest, which were 40 and 30 respectively. The reference ratios of the two methods were the same soil background value, so the difference of toxicity coefficients leads to great changes in EI_*i*_. Compared with I_geo_, the EI_*i*_ considers not only the content of HMs, but also the biological toxicity of different metals^34,35^.

### Sources of HMs in agricultural soils

#### Correlation analysis

Correlation tests were used to understand the relationship between different HMs and find their possible sources^36^. If there is a positive correlation between HMs may be indicative of their common source. As shown in Table 3, the most of HMs exhibited significant correlations(P < 0.05). Especially, Hg-Cd, As-Cd, Cr-Pb, Cu-Cd, Ni-Hg-Pb, Ni-Cu and Zn-Cr-Ni exhibited high significant correlations(P < 0.01), which meaning that these HMs in the study area may have common origin. Moreover, significant correlations were also observed between pH with Cd, Pb, Ni and Cr, TN with Cd, Hg, As and Pb, TP with Cd, As and Cu, TK with Hg, As, Pb, Cr and Ni, OM with Cd, Cr, Hg, As, Pb and Cu, which indicate that the source of HMs in the study area may be closely related to human activities.

**Table 3:** Results of Pearson’s correlation analysis of HMs.

	Cu	Pb	Zn	Cr	Ni	Cd	As	Hg	TP	TK	TN	OM	pH
Cu	1												
Pb	0.051	1											
Zn	0.033	0.076*	1										
Cr	− 0.004	0.219**	0.213**	1									
Ni	0.250**	0.290**	0.314**	0.454**	1								
Cd	0.386**	0.148**	0.069	0.019	0.039	1							
As	− 0.053	− 0.072*	− 0.072*	− 0.014	0.086*	− 0.312**	1						
Hg	0.091*	0.602**	0.087*	0.087*	0.191**	0.161**	− 0.129**	1					
TP	0.882**	− 0.010	0.007	− 0.069	0.051	0.462**	− 0.199**	0.052	1				
TK	− 0.043	0.197**	0.038	0.370**	0.585**	− 0.076*	− 0.140**	0.156**	− 0.064	1			
TN	0.023	0.146**	0.010	0.046	− 0.016	0.103**	− 0.137**	0.123**	0.038	0.035	1		
OM	0.143**	0.364**	0.021	0.114**	− 0.033	0.599**	− 0.482**	0.236**	0.286**	0.002	0.245**	1	
pH	0.032	− 0.338**	0.004	− 0.129**	− 0.103**	0.272**	0.045	− 0.028	− 0.025	− 0.182**	− 0.104**	− 0.138**	1

*Shows significant correlation at the 0.05 level (2-tailed).

**Shows significant correlation at the 0.01 level (2-tailed).

#### Principal component analysis

Principal component analysis (PCA) is used widely for analyzing the sources of soil HMs. Four factors were extracted by maximum variance rotation method and the results are shown in Table 4. The initial eigenvalues of principal component 1 (PC-1), principal component 2 (PC-2), principal component 3 (PC-3) and principal component 4 (PC-4) were greater than 1, and explaining cumulative variance of four PC were 73.416%. PC-1 consists of Pb and Hg, accounted for 20.637% of the total variance. The mean concentrations of Pb and Hg in soil were 32.2 mg kg^−1^ and 0.155 mg kg^−1^, significantly higher than the soil background value for Chengdu (Table 1). High value points for Pb and Hg were mainly concentrated in TangChang town government and surrounding residential areas. It seems that anthropogenic activities sources such as traffic and atmospheric subsidence were the major sources of Pb and Hg in agricultural soils. PC-2 consists of Zn, Cr and Ni, accounted for 20.446% of the total variance. These results were consistent with the results of Pearson’s correlation analysis (Table 3). Although many researchers have found that these elements had some degree of homology and may be affected mainly by material and pedogenic processes^30,31,37^, but their mean concentrations higher than the soil background value for Chengdu. Therefore, the natural sources and industrial activities together constituted the major sources of PC-2. PC-3 consists of Cu and Cd, accounted for 17.063% of the total variance. Very significant correlation was found with Cu-P and Cd-P. The planting of hotbed chives needs to use a lot of phosphate fertilizer. Therefore, it seems that long-term use of chemical fertilizers and pesticides was the major source of PC-3. PC-4 includes only As, accounted for 15.271% of the total variance. The As in the soil of the whole town was generally in a pollution-free state. It seems that the natural sources were the major sources of PC-4 and many studies have reached similar conclusions^38,39^.

**Table 4:** Results of the principal component analysis for HMs.

Component	Cu	Pb	Zn	Cr	Ni	Cd	As	Hg	Initial eigenvalues	Explained variance (%)	Explained of cumulative variance (%)
PC-1	0.011	**0.883**	− 0.074	0.139	0.232	0.126	− 0.079	**0.878**	1.651	20.637	20.637
PC-2	0.049	0.162	**0.713**	**0.747**	**0.735**	− 0.013	− 0.017	0.032	1.636	20.446	41.082
PC-3	**0.917**	0.029	− 0.034	− 0.054	0.287	**0.657**	− 0.034	0.061	1.365	17.063	58.145
PC-4	0.066	− 0.011	− 0.264	0.049	0.277	− 0.514	**0.891**	− 0.103	1.222	15.271	73.416

Significant values are in bold.

### Health risk assessment

#### Non-carcinogenic risk assessment

**Figure 3:**
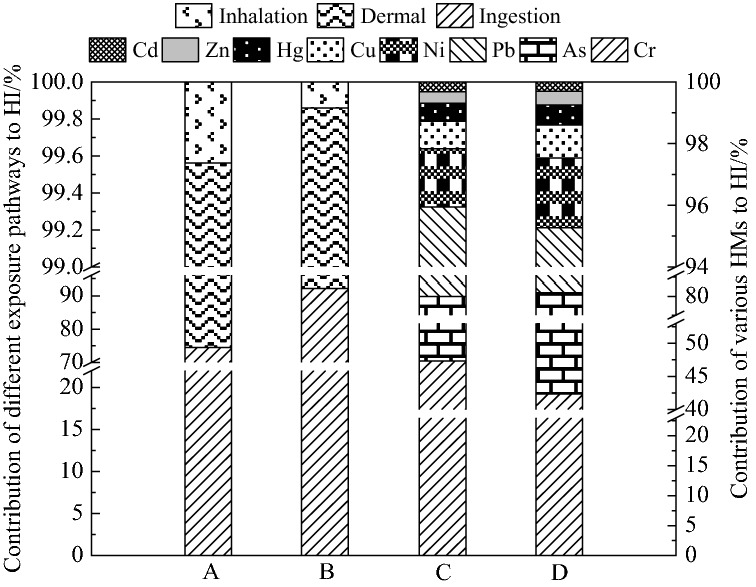
Contribution of different exposure pathways and various HMs to hazard index. A stand for HI of adults and B for HI of children by contribution of different exposure pathways, C stand for HI of adults and D for HI of children by contribution of various HMs.

Table 5 and Fig. 3 show the results for non-carcinogenic risk. In collected soil samples the HQ value of three exposure pathways (ingestion, dermal contact, and inhalation) for eight HMs were lower than 1. The HI for adults and children were 0.173 and 0.996, respectively. Suggesting no significant non-carcinogenic risk in the study soils. It is noteworthy that the HI of children was higher than the HI of adults, meaning that children were more susceptible to adverse effects. Other studies have shown similar phenomenon^1,2,36^. The reason may be children's special behavior and physiological characteristics, such as frequent pica behavior and hand or finger sucking and so on^40,41^.

**Table 5:** Statistics analysis for non-carcinogenic risk index and carcinogenic risk index of HMs.

		Cd	Hg	As	Pb	Cr	Cu	Ni	Zn	THI	TCR
HQ_ing_	Adults	3.74E−04	8.70E−04	5.49E−02	1.56E−02	5.17E−02	1.48E−03	3.13E−03	6.12E−04	1.29E−01	–
	Children	2.67E−03	6.22E−03	3.93E−01	1.11E−01	3.70E−01	1.06E−02	2.24E−02	4.37E−03	9.19E−01	–
HQ_inh_	Adults	3.98E−06	3.25E−07	5.86E−06	1.65E−04	5.78E−04	1.58E−07	3.24E−06	6.53E−08	7.57E−04	–
	Children	7.36E−06	6.00E−07	1.08E−05	3.05E−04	1.07E−03	2.91E−07	5.99E−06	1.21E−07	1.40E−03	–
HQ_derm_	Adults	1.70E−04	1.42E−04	1.53E−03	1.18E−02	2.95E−02	5.64E−05	1.32E−04	3.49E−05	4.34E−02	–
	Children	2.99E−04	2.49E−04	2.68E−03	2.07E−02	5.17E−02	9.90E−05	2.32E−04	6.12E−05	7.61E−02	–
THI	Adults	5.48E−04	1.01E−03	5.65E−02	2.75E−02	8.18E−02	1.54E−03	3.27E−03	6.47E−04	1.73E−01	–
	Children	2.98E−03	6.46E−03	3.95E−01	1.32E−01	4.22E−01	1.07E−02	2.26E−02	4.43E−03	9.97E−01	–
CR_ing_	Adults	7.91E−07	8.59E−06	1.61E−07	2.69E−05	–	–	–	–	–	3.65E−05
	Children	1.36E−06	1.47E−05	2.75E−07	4.62E−05	–	–	–	–	–	6.25E−05
CR_inh_	Adults	8.71E−11	9.21E−09	8.46E−11	2.41E−07	1.95E−09	–	–	–	–	2.53E−07
	Children	3.86E−11	4.09E−09	3.75E−11	1.07E−07	8.63E−10	–	–	–	–	1.12E−07
CR_derm_	Adults	9.01E−09	9.79E−08	–	–	–	–	–	–	–	1.07E−07
	Children	3.80E−09	4.12E−08	–	–	–	–	–	–	–	4.50E−08
CR	Adults	8.00E−07	8.69E−06	1.61E−07	2.72E−05	1.95E−09	–	–	–	–	3.68E−05
	Children	1.36E−06	1.48E−05	2.76E−07	4.63E−05	8.63E−10	–	–	–	–	6.27E−05

From Fig. 3A,B, It was easy to show that the contribution of different pathways to non-carcinogenic risk was similar between adults and children decreased in the following order: Ingestion > Dermal contact > Inhalation. The HI mean values for adults and children by ingestion, dermal contact and inhalation routes were 0.129 and 0.919, 0.0434 and 0.0761, 0.000757 and 0.0014, respectively. It's not hard to saw the HI for ingestion route of both adults and children were 1–3 orders of magnitude higher than the other two exposure pathways. Many studies have reached similar conclusions^32,36^. Thus, the ingestion route may be an important pathway for HMs exposure in study area.

The HI mean value of single HM for children and adults decreased as following order (Fig. 3C,D): Cr > As > Pb > Ni > Cu > Hg > Zn > Cd. Cr, As and Pb were the largest contributors for both adults and children, accounting for 47.33% and 42.37%, 32.68% and 39.64%, 15.95% and 13.26%, respectively. Indicating that attention should be pain to the Cr, As and Pb elements due to their noncarcinogenic risk. Which was basically consistent with the research results of Bo et al.^1^ and Bao et al.^32^. Overall, The HMs of Cr, As and Pb were the main non-carcinogenic factors in soil in the study area, and the risk control of these elements should be strengthened.

#### Carcinogenic risk assessment

The CR of the HMs are shown in Table 5. Three exposure pathways were considered for Cd and Hg in our study. But As and Pb were considered carcinogenic by ingestion and inhalation, and Cr was considered carcinogenic through inhalation. The TCR mean values were 3.68 × 10^−5^ for adults and 6.27 × 10^−5^ for children, obviously were in the range 1 × 10^−4^ from 1 × 10^−6^, suggesting the TCR caused by HMs in the study area was acceptable on the whole, but it still exceeds the soil treatment threshold value 10^−6^. The CR average values through the three exposure pathways were CR_ing_ 3.65 × 10^−5^, CR_inh_ 2.53 × 10^−7^ and CR_derm_ 1.07 × 10^−7^ for adults, CR_ing_ 6.25 × 10^−5^, CR_inh_ 1.12 × 10^−7^ and CR_derm_ 4.50 × 10^−8^ for adults. Clearly the CR_ing_ was much larger than CR_inh_ and CR_derm_ for both adults and children, which indicates that oral ingestion is the major exposure pathway for CR. Which was consistent with the research results of Song et al.^31^ and Bo et al.^1^. For single HM, the CR value of Pb and Hg for adults were 2.72 × 10^−5^ and 8.6 × 10^−6^, and Pb, Hg and Cd for children were 4.63 × 10^−5^, 1.48 × 10^−5^ and 1.36 × 10^−6^, respectively, within acceptable criterion. Overall, the longterm health effects for adults and children are not serious at current single HM level.

In this study, the total contents of HMs in the soils were used to assess health risk, the bioavailability of HMs were not considered, which may have caused the assessment results to be higher than the actual local situation^1,31,42^. In addition, because the parameters of health risk evaluation for children were set to be more sensitive than those for adults, the non-carcinogenic risk and carcinogenic risk for children were higher than those for adults^2,43,44^. However, those risks were at acceptable or negligible levels. Therefore, the study area is suitable for safe and clean production of hotbed chives.

## Conclusion

In the study area, the average contents of HMs Cd, Hg, Ni, As, Cu, Cr, Pb and Zn in the soil do not exceed the threshold values of the risk screening values for soil contamination, and are generally at a clean level. In addition to natural sources, Pb and Hg may mainly come from transportation and atmospheric deposition, Zn, Cr and Ni may mainly come from industrial activities, and Cu and Cd may mainly come from agricultural activities such as fertilization, pesticide application and irrigation. In general, the potential ecological risk of soil HMs is low, but a few areas had a moderate risk or considerable risk. Overall, the HMs of Cr, As and Pb are the main non-carcinogenic factors in soil in study area. Although there is no significant human health risk, however, the non-carcinogenic risk and carcinogenic risk for children are higher than those for adults. In order to reduce the content and human health risks of heavy metal(loid)s in agricultural soils, ensure the green and sustainable production capacity of the soil in hotbed chives production area, strengthening soil management, reducing the use of pesticides and chemical fertilizers, surface water irrigation, improving soil quality monitoring and removalling dust treatment of relevant industrial activities are good long-term solutions.
